# Recent Advances in Developing Artificial Autotrophic Microorganism for Reinforcing CO_2_ Fixation

**DOI:** 10.3389/fmicb.2020.592631

**Published:** 2020-11-09

**Authors:** Bo Liang, Yukun Zhao, Jianming Yang

**Affiliations:** ^1^Energy-rich Compounds Production by Photosynthetic Carbon Fixation Research Center, Qingdao Agricultural University, Qingdao, China; ^2^Shandong Key Lab of Applied Mycology, College of Life Sciences, Qingdao Agricultural University, Qingdao, China; ^3^Pony Testing International Group, Qingdao, China

**Keywords:** CO_2_ fixation, autotrophy, heterotrophy, synthetic biology, reducing power, cell factory

## Abstract

With the goal of achieving carbon sequestration, emission reduction and cleaner production, biological methods have been employed to convert carbon dioxide (CO_2_) into fuels and chemicals. However, natural autotrophic organisms are not suitable cell factories due to their poor carbon fixation efficiency and poor growth rate. Heterotrophic microorganisms are promising candidates, since they have been proven to be efficient biofuel and chemical production chassis. This review first briefly summarizes six naturally occurring CO_2_ fixation pathways, and then focuses on recent advances in artificially designing efficient CO_2_ fixation pathways. Moreover, this review discusses the transformation of heterotrophic microorganisms into hemiautotrophic microorganisms and delves further into fully autotrophic microorganisms (artificial autotrophy) by use of synthetic biological tools and strategies. Rapid developments in artificial autotrophy have laid a solid foundation for the development of efficient carbon fixation cell factories. Finally, this review highlights future directions toward large-scale applications. Artificial autotrophic microbial cell factories need further improvements in terms of CO_2_ fixation pathways, reducing power supply, compartmentalization and host selection.

## Introduction

The carbon element is the most important component in all types of living organic matter, accounting for approximately 50% of the dry weight of the organics. In nature, elemental carbon exists in many forms, including carbon dioxide (CO_2_) in the atmosphere, CO_2_ dissolved in water (H_2_CO_3_, HCO${}_{3}^{-}$, and CO${}_{3}^{2-}$) and carbon in organics, as well as carbon in rocks and fossil fuels. Currently, combustion of fossil fuels leads to considerable emission of greenhouse CO_2_ and causes global warming, which is a major concern for all societies (Irfan et al., 2019). Thus, it is urgent to minimize CO_2_ emissions by reducing the consumption of fossil fuels and reinforcing CO_2_ fixation (Pacala and Socolow, 2004). Compared to chemical methods, biological carbon sequestration is an attractive option for CO_2_ fixation, as it has several advantages, including mild reaction conditions and an eco-friendly approach (Li et al., 2012). Organisms that fix carbon include plants and autotrophic microorganisms. In green plants, carbon flux occurs primarily from CO_2_ to biomass driven by solar energy. Autotrophic microorganisms exist in certain special conditions on Earth in which plants cannot live, and they assimilate CO_2_ into biomass driven by solar energy or chemical energy produced by oxidizing inorganic substance in a more direct and rapid way. It is supposed that CO_2_ fixation might be more economic and efficient when sustainable bioprocesses producing biofuel and valuable chemicals directly from CO_2_ are realized. In this respect, autotrophic microorganisms can be regarded as the most suitable cell factories (Liu et al., 2020).

Autotrophic microorganisms are capable of incorporating CO_2_ into biomass via six natural carbon fixation pathways (Bar-Even et al., 2012). Since the discovery of the Calvin-Benson-Bassham (CBB) cycle in the 1940s and 1950s, another five CO_2_ assimilation mechanisms have been elucidated, namely, the reductive citric acid cycle (rTCA), the reductive acetyl-CoA pathway (Wood-Ljungdahl pathway), the 3-hydroxypropionate bicycle (3HP bicycle), the 3-hydroxypropionate/4-hydroxybutyrate cycle (3HP/4HB cycle), and dicarboxylate/4-hydroxybutyrate cycle (DC/HB cycle) (Berg, 2011). The CBB cycle is ubiquitous in autotrophic microorganisms, primarily occurring in cyanobacteria. Remarkable progress has been made in producing biofuel and chemicals from CO_2_ and solar energy by engineering the native CBB cycle in cyanobacteria, including ethanol, butanol, lactic acid, acetone, isobutyraldehyde, isoprene, and biodiesel (Savakis and Hellingwerf, 2015; Veaudor et al., 2020). However, the titer of target products is unsatisfactory (Hagemann and Hesse, 2018; Luan et al., 2020). The CO_2_ capturing rate of ribulose-1,5-bisphosphate carboxylase/oxygenase (Rubisco) (EC 4.1.1.39) in the CBB cycle is an order of magnitude lower than the average of central metabolic enzymes, and efforts to improve Rubisco’s kinetic properties have attained only limited success so far (Antonovsky et al., 2017; Liang et al., 2018). Moreover, low levels of growth and CO_2_ fixation efficiency, as well as deficient genetic operating platforms have largely limited industrial applications of cyanobacteria (Blankenship et al., 2011; Kushwaha et al., 2018). Hence, there is a need for faster and more efficient bioprocesses for the conversion of CO_2_ into the desired products. Generally, heterotrophic microorganisms have the advantage that growth and production yields are typically superior compared to the autotrophic life cycle. Massive and mature genetic manipulation tools make heterotrophy more accessible to a brand-new metabolism and proliferation mode (Nielsen and Keasling, 2016). The considerable advances in synthetic biology allow the engineering of novel functions and *de novo* metabolism networks in heterotrophic microorganisms for industrial biotechnology applications (Chen Y. et al., 2020; Clarke and Kitney, 2020). Previous developments in engineering of CO_2_-fixing pathways for improving the efficiency of CO_2_ fixation in autotrophic and heterotrophic microorganisms have been addressed in reported reviews (Claassens et al., 2016; Antonovsky et al., 2017; Hu et al., 2018).

**FIGURE 1:**
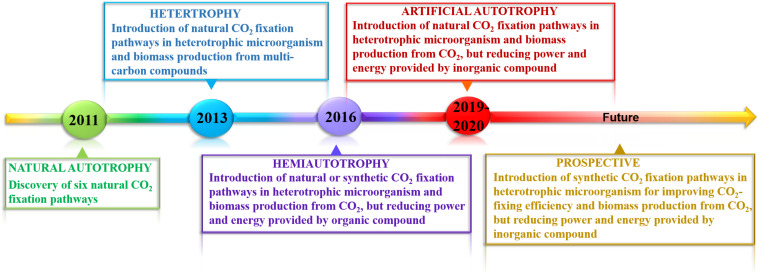
Milestone in developing artificial autotrophy in the last decade. Until 2011, six naturally occurring CO_2_ fixation pathways have been identified, some of which were introduced into heterotrophic microorganisms. However, all of biomass was derived from additional multi-carbon compounds, such as glucose. The strong advances in synthetic biology enable the engineering of synthetic CO_2_ fixation pathways for improving carbon assimilation efficiency. Since 2016, hemiautotrophy has been successfully constructed by integrating natural or synthetic CO_2_ fixation pathways in heterotrophic hosts using organic compound as reducing power and energy source, in which biomass was completely derived from CO_2_. In 2019 and 2020, the conversion of heterotrophy to fully autotrophy was realized though integration of natural CO_2_ fixation pathways to support cell growth and inorganic compound’s oxidation to provide reducing power and energy.

In this review, we discuss recent advances in developing artificial autotrophic microorganisms for reinforcing CO_2_ fixation (Figure 1). Starting from heterotrophs to hemiautotrophs, and finally to complete autotrophs, a variety of natural and artificial synthetic CO_2_ fixation pathways have been developed and introduced into heterotrophic chassis. These engineered artificial autotrophs represent very promising candidates for highly efficient CO_2_ fixation and sustainable production of biofuels and value-added chemicals.

## Natural Co_2_ Fixation Pathways

Carbon dioxide assimilation is the process of reducing CO_2_ into cellular carbon which requires reducing equivalents and energy provided by ATP hydrolysis. The reductant varies by different microorganisms. In anaerobes, low-potential electron donors bearing more energy are responsible for offering reducing equivalents, such as ferredoxin E_0_’ ≈ −400 mV. In contrast, aerobic microbial organisms require more ATP equivalents since high-potential electron donors with less energy are used as electron donors, such as NADPH E_0_’ ≈ −320 mV (Berg, 2011). The sensitivity of enzymes toward molecular oxygen decides the distribution of CO_2_ fixation pathways in both anaerobic and aerobic microorganisms.

Currently, six autotrophic CO_2_-fixing pathways in microorganisms have been elucidated in detail. These pathways are divided into two groups according to the tolerance of key enzymes toward oxygen. The aerobic pathways include the CBB cycle, 3HP bicycle and 3HP/4HB cycle, while the rTCA cycle, Wood-Ljungdahl pathway and DC/HB cycle belong to the anaerobic pathways group since strictly anaerobic enzymes operate in these routes.

For common microorganisms, the CBB cycle is the most important mechanism of autotrophic CO_2_ fixation (Bar-Even et al., 2012). The entire cycle is composed of 13 steps and three stages, consisting of carboxylation, reduction and regeneration (Bassham and Calvin, 1962). First, the key enzyme Rubisco catalyzes the carboxylation of CO_2_ and ribulose-1,5-bisphosphate (RuBP) to generate 3-phosphoglycerate (3-PGA). Free energy change (Δ_r_Gm′) is −37.8 kJ/mol. Then this product is reduced to glyceraldehyde-3-phosphate (GAP) consuming ATP and NADPH by 3-phosphoglycerate kinase and GAP dehydrogenase, in which the reaction catalyzed by 3-phosphoglycerate kinase is thermodynamically challenging (Δ_r_Gm′ = +18.7 kJ/mol). Next, 5-bisphosphate (Ru5P) is regenerated through conversion between C_3_, C_4_, C_5_, C_6_, and C_7_ sugar. Finally, Ru5P is phosphorylated by phosphoribulokinase to regenerate RuBP (Δ_r_Gm′ = −24.2 kJ/mol). After one cycle, the CBB cycle can fix three CO_2_ molecules and produce one GAP molecule at the cost of nine molecules of ATP and six molecules of NADPH reducing equivalents. The regeneration of energy carrier (ATP) and reducing equivalents (NADPH) in living microbes can be realized by photosystems. Despite the CBB cycle being the most prevalent CO_2_ fixation mechanism on Earth, the efficiency of carbon assimilation is unsatisfactory. First, the final product of this cycle is a C3 compound that is inefficient for the synthesis of acetyl-CoA since CO_2_ is inevitably lost during the conversion of GAP to acetyl-CoA. Acetyl-CoA is the central precursor for producing value-added multicarbon compounds, such as fatty acids (Blatti et al., 2013). Furthermore, the strong side reaction of Rubisco with oxygen can incur loss of fixed carbon, and the side product 2-phosphoglycolate is toxic to cell (Hagemann and Bauwe, 2016). In addition, large amounts of ATP and NADPH are consumed in this cycle (Berg, 2011).

As the key enzyme in the CBB cycle, Rubisco is the most abundant protein in the biosphere, which fixes ∼10^11^ tons of CO_2_ from the atmosphere per year (Hayer-Hartl and Hartl, 2020). Rubisco is a well-studied enzyme in photosynthetic organism since it provides organic carbon for life (Iniguez et al., 2020). Crystal structures of Rubisco from several origins have been analyzed by X-ray, including *Rhodospirillum rubrum*, *Synechococcus* PCC6301, *Chlorobium tepidum*, *Thermococcus kodakaraensis* KOD1, and so on (Iniguez et al., 2020). Two forms of Rubisco participate in the CBB cycle. Form I exists in all photosynthetic organisms, consisting of eight large-subunits (50–60 kDa) and eight small-subunits (12–18 kDa) that form an L8S8 structure. The tertiary structure of Form I is similar to a barrel, in which the L8 core is formed through the tetramerization of four interactional L2 dimers. Eight small subunits equally divided into two sets separately assemble the L8 core upside and downside. The large subunit harbors catalytic sites, especially amino acid sequences between 169 and 220, as well as 321 and 340. Compared to the small subunits, homology of the large subunits among different species is high. Small subunits have regulatory functions. Form II Rubisco is found in purple nonsulfur photosynthetic bacteria, such as *Rhodospirillum rubrum* and some marine dinoflagellates. This type is composed of two large-subunits (L2). Form I and Form II Rubisco are simultaneously found in several nonsulfur phototropic bacteria, such as *Rhodobacter sphaeroides* and *R. capsulatus*, as well as *Thiobacillus* sp. and *Hydrogenovibrio marinus*. Although Rubisco plays a pivotal role in global carbon fixation, its extremely poor carboxylation activity and competing oxygenase activity greatly hinder high carbon-fixing efficiency in the CBB cycle. CO_2_ and O_2_ competitively combine in the active site of Rubisco. The competitive efficiency for CO_2_ and O_2_ is defined as the specificity factor (Ω = *V*_c_*K*_o_/*V*_o_*K*_c_), which is the ratio of the catalytic efficiency (*V*_max_/*K*_M_) for the carboxylation and oxygenation reactions. The specificity factor Ω of Form II (Ω = 10 to15) is much lower than that of Form I (Ω = 25 to 75 in bacteria). Significant efforts have been made to improve the value of specificity factor Ω and the efficiency of carbon capturing. In recent years, remarkably improved heterologous expression levels have been realized. However, molecular modification of Rubisco has been challenging owing to its complicated protein structure and intricate nature. *Escherichia coli* has long been the preferred host for the directed evolution of Rubisco for the sake of enhancing carboxylation activity toward CO_2_ due to its high transformation efficiency and simple genetic tools (Mueller-Cajar and Whitney, 2008a). Several screening platforms for directed evolution of Rubisco have been developed by coupling cell growth to Rubisco activity (Parikh et al., 2006; Mueller-Cajar et al., 2007; Mueller-Cajar and Whitney, 2008b; Cai et al., 2014; Wilson et al., 2016; Zhou and Whitney, 2019). Unfortunately, affinity and catalytic efficiency toward CO_2_ failed to synchronously increase (Kubis and Bar-Even, 2019). Moreover, remarkable kinetic enhancement was not obtained, likely due to an improper library screening platform, such as the host. In addition to model heterotrophs, *Rhodobacter capsulatus* has been used as an autotrophic host for directed evolution and rational design of Rubisco (Satagopan et al., 2019). In the *R. capsulatus* Rubisco deletion strain, complementation of a foreign Rubisco gene allowed the selection of substitutions. These screened mutants exhibited an improved affinity toward CO_2_ rather than improved carboxylation activity. For example, the V186I/T327A^L^ mutant showed a 42% decreased *K*_c_ and a 41% increased *K*_o_. These mutant sites are located in the hemiconserved hydrophobic region adjacent to the active site and connect the large-small interface. The above studies related to Rubisco engineering are limited to bacterial Rubisco since prokaryotic hosts cannot form the eukaryotic holoenzyme. By swapping certain residues of the large-subunit’s primary structure that are responsible for preventing heterologous eukaryotic holoenzyme formation, Koay et al. (2016) created bacterial and eukaryotic Rubisco hybrids that could express and assemble in *E. coli*. These chimeric Rubisco proteins serve as targets for directed evolution and rational design in the future work.

The 3HP bicycle was first discovered in 1986 by Helge Holo and primarily exists in photosynthetic green non-sulfur bacteria, such as *Chloroflexus* (Mattozzi et al., 2013). In the first cycle, three HCO${}_{3}^{-}$ molecules and one acetyl-CoA molecule are converted to glyoxylate via ten steps catalyzed by ten enzymes (Δ_r_Gm′ = −109.4 kJ/mol). In the second cycle, glyoxylate and propionyl-CoA synthesize acetyl-CoA and pyruvate through five steps (Δ_r_Gm′ = −55.4 kJ/mol). In addition to the number of steps in this pathway, the energy costs are higher than in the other four natural pathways. 3HP bicycle fixes three CO_2_ molecules and produces one pyruvate molecule with the consumption of seven ATP molecules and five molecules of reducing equivalents. A “chimeric” photosynthetic system is used to regenerate ATP and reducing equivalents (Xin et al., 2018).

The 3HP/4HB cycle was first identified in archaea (Berg et al., 2007). In this pathway, acetyl-CoA/propionyl-CoA carboxylase fixes two molecules of HCO${}_{3}^{-}$ to produce succinyl-CoA (Δ_r_Gm′ = −61.9 kJ/mol). To regenerate acetyl-CoA, succinyl-CoA is reduced to 4-hydroxybutyrate, which is then activated to 4-hydroxybutyryl-CoA (Δ_r_Gm′ = −17.0 kJ/mol). Crotonyl-CoA is synthesized by the key enzyme 4-hydroxybutyryl-CoA dehydratase from 4-hydroxybutyryl-CoA (Δ_r_Gm′ = −7.7 kJ/mol). Finally, crotonyl-CoA is oxidized and subsequently cleaved into acetyl-CoA (Δ_r_Gm′ = −16.5 kJ/mol). The 3HP/4HB cycle fixes two molecules of HCO${}_{3}^{-}$ to produce one molecule of acetyl-CoA at the expense of six ATPs and four NAD(P)H reducing equivalents after 16 steps. In this cycle, the hydrolysis of pyrophosphate may provide energy (Berg, 2011).

The rTCA cycle exists in photosynthetic green sulfur bacteria and anaerobic bacteria (Buchanan and Arnon, 1990). This cycle reverses the reactions of the oxidative citric acid cycle (TCA cycle) and forms acetyl-CoA from two molecules of CO_2_ at the cost of two molecules of ATP, which is far less than the above three pathways. The regeneration of ATP and reducing equivalents in living microbes can be realized by photosynthetic reaction center type I (Berg, 2011). To reverse the TCA cycle, three irreversible enzymes are required, including fumarate reductase, ferredoxin-dependent 2-oxoglutarate synthase and ATP-citrate lyase, among which ferredoxin-dependent 2-oxoglutarate synthase is strictly anaerobic. Thermodynamically challenging reactions (Δ_r_Gm′ > 10 kJ/mol) are catalyzed by ATP-citrate lyase, 2-ketoglutarate synthase and isocitrate dehydrogenase in this cycle.

The Wood-Ljungdahl pathway is characterized by high energetic efficiency because in this route two CO_2_ molecules are fixed to produce acetyl-CoA with the consumption of only one ATP (Ljungdahl, 1969). In detail, this pathway begins with one CO_2_ molecule reduction to formic acid by highly oxygen sensitive formate dehydrogenase (FDH), which is thermodynamically challenging (Δ_r_Gm′ = +18.0 kJ/mol). Another CO_2_ molecule is reduced to carbon monoxide by a nickel atom in the active center of another highly oxygen sensitive CO dehydrogenase, which is also thermodynamically challenging (Δ_r_Gm′ = +32.6 kJ/mol). Then formic acid is attached to tetrahydrofolate (THF) for further reduction. Finally, the one-carbon unit transfers to nickel-bound CO and forms acetyl-CoA. In acetogens, CO_2_ is reduced to acetic acid with H_2_ via the Wood-Ljungdahl pathway, in which the ATP required for formate activation is regenerated in the acetate kinase reaction (Mock et al., 2015; Lemaire et al., 2020).

The DC/HB cycle is a another strictly anaerobic CO_2_ fixation pathway, in which two molecules of HCO${}_{3}^{-}$ and acetyl-CoA are converted to succinyl-CoA by two carboxylase pyruvate synthase and phosphoenolpyruvate (PEP) carboxylase (Huber et al., 2008). The regeneration of acetyl-CoA occurs through the same route as in the 3HP/4HB cycle. However, some enzymes and electron carriers, such as pyruvate synthase and ferredoxin, are inactivated by oxygen. Within this cycle, one molecule of HCO${}_{3}^{-}$ and one molecule of CO_2_ are fixed to generate one molecule of acetyl-CoA via 13 steps catalyzed by 10 enzymes with the consumption of five molecules of ATP.

## Introduction of Natural Co_2_ Fixation Pathways Into Heterotrophic Microorganisms

Although autotrophic organisms are the sponsors to fix CO_2_ in nature, low activity of carbon capturing dramatically influences the efficient conversion of CO_2_ to biomass. Many efforts have been made to improve CO_2_ carboxylation rate during the CBB cycle since Rubisco exhibits an extremely low affinity toward CO_2_ and obvious oxygenation side-reactions in conditions of low levels of CO_2_ (Ducat and Silver, 2012; Lin et al., 2014). Unfortunately, limited success has been achieved through engineering Rubisco or other related enzymes. Moreover, it is challenging to attempt genetic manipulation of autotrophic microorganisms owing to their unclear genetic background and the absence of genetic tools. Meanwhile, overexpressing new genes responsible for foreign CO_2_ fixation pathway in autotrophic microorganisms may exert negative influences on regulatory networks in cells, such as carbon/nitrogen metabolism (Song et al., 2020). As model heterotrophs, *E. coli* and *Saccharomyces cerevisiae* have been widely used to serve as chassis to express natural CO_2_ fixation routes (Antonovsky et al., 2017).

### E. coli

The 3-HP bi-cycle from *Chloroflexus aurantiacus* has been successfully integrated into *E. coli*. To heterologously express the entire bicycle, a grouping strategy was employed, by which a 16-step enzymatic reaction was divided into four functional subpathways, and each group was independently expressed in a synthetic operon. This study provides evidence for the evolution of bacteria by horizontal gene transfer (Mattozzi et al., 2013). However, the functional co-expression of the overall bicycle in *E. coli* failed to support autotrophic growth.

The possibility of producing sugar for cell proliferation was explored by switching off the inflow of organic carbon into the CBB cycle. In this pioneer work, reconstruction of the fully functional CBB cycle conferred in *E. coli* the capacity to synthesize sugar and other biomass components from CO_2_ by implementing a comprehensive approach that involved rational metabolic network construction, heterologous recombination expression and adaptive laboratory evolution (ALE). To cut off gluconeogenesis, central metabolism was divided into two independent modules by introducing Rubisco from *Synechococcus* sp. PCC 7002 and phosphoribosyl kinase (PRK) from *S. elongatus* PCC 7942, simultaneously deleting the phosphoglycerol mutase gene. The first module included upstream glycolysis, pentose phosphate pathway (PPP) and recombinant CBB cycle enzymes (Rubisco and PRK). Meanwhile, the second module was an energy portion containing downstream glycolysis and TCA cycle, providing ATP and reducing power for carbon fixation of module one. However, the resultant strain could not grow in the presence of CO_2_ and pyruvate (In this study, energy and reducing power were supplied by the oxidation of pyruvate). Only addition of a second sugar could support cell growth. The ALE strategy has advanced our ability to rapidly obtain a desired phenotype (Lee and Kim, 2020). The engineered strain was initially grown in a xylose-restricted chemostat, and at last, the evolved strain could grow with pyruvate as the sole carbon source at high CO_2_ concentrations. Further mass spectrometry analysis showed that CO_2_ was the only carbon source for phospho-sugars synthesis in the evolved strain, including ribose-5 phosphate, sedoheptulose-7 phosphate, fructose-6-phosphate, and glucose-6 phosphate, indicating that the fully functional CBB cycle in *E. coli* can directly synthesize sugar from CO_2_. Four molecules of pyruvate were consumed per sugar. After genome-wide sequencing, the *prs* gene, encoding ribose phosphate pyrophosphate kinase, was the only common mutant gene found in three chemostat experiments, which is the primary branching enzyme of the CBB cycle module. This study shows that the functionality of the CBB cycle depends not only on heterologous enzymes (Rubisco and PRK) but also on the endogenous components that interact with them, particularly metabolic enzymes in the circulating carbon pool (Antonovsky et al., 2016). Proper balance of kinetic properties in enzymes at branch point is imperative to maintain a stable metabolism *in vivo* (Barenholz et al., 2017). In this work, three molecules of CO_2_ were fixed to one molecule of pyruvate via CBB cycle. However, three molecules of CO_2_ were produced during the TCA cycle using pyruvate as substrate. Therefore, the net CO_2_ fixation was zero.

### S. cerevisiae

Biofuel has been widely regarded as a promising alternative to fossil fuels concerning energy security, renewability, and global warming (Caspeta et al., 2013). Bioethanol is currently the most highly produced biofuel on an industrial scale (Nielsen et al., 2013). *S. cerevisiae* is the most commonly used host for bioethanol fermentation due to its high ethanol productivity and strong ethanol tolerance (Alper et al., 2006). However, during the process of yeast fermentation to produce ethanol, the production of ethanol is accompanied by CO_2_ release, causing carbon loss and greenhouse gas emissions. In addition, excessive NADH causes excessive accumulation of the byproduct glycerol. To recycle CO_2_, heterologous PRK and Rubisco derived from various origins were introduced into *S. cerevisiae* to construct the CO_2_-fixing route. The product of CO_2_ fixing by Rubisco is glycerate 3-phosphate (G3P). Ethanol is produced from G3P. At the same time, glycerol can also be formed using GAP as substrate by the actions of glycerol-3-phosphate dehydrogenase and glycerol-3-phosphatase. GAP is produced during xylose fermentation via non-oxidative PPP. This approach increased ethanol production and reduced the accumulation of the byproduct glycerol. For example, researchers co-expressed PRK from *spinach* and Rubisco from *Thiobacillus denitrificans* in *S. cerevisiae*. *T. denitrificans* Rubisco belongs to type II, composed of 8 large subunits. Its active expression requires the assistance of *E. coli* chaperons GroEL and GroES. Compared to the original strain, 90% in the reduction of byproduct glycerol and 10% increase of the production of ethanol were realized when the media was supplemented with glucose and galactose (Guadalupe-Medina et al., 2013). The use of sugarcane and corn starch as raw materials for the production of ethanol has the problem of “competing with people for food” (Tilman et al., 2009). As the second most abundant sugar in hydrolysis products from lignocellulose, xylose is an ideal feedstock to yield bioethanol via the xylose reductase (XR)/xylitol dehydrogenase (XDH) pathway in *S. cerevisiae* (Nogue and Karhumaa, 2015). CO_2_ fixation has been achieved by introducing PRK from *Spinacia oleracea* and Rubisco from *Rhodospirillum rubrum* using xylose as substrate. Three molecules of CO_2_ were fixed into one molecule of GAP by introducing PRK and Rubisco into yeast. Then, one molecule of ethanol was produced from one molecule of GAP with releasing one molecule of CO_2_. And pyruvate was formed from GAP to produce acetyl-CoA for other metabolism to support cell growth with inevitably releasing CO_2_. Therefore, the net production of CO_2_ was observed in this engineered strain. Note that, results showed that the reduced release of CO_2_ was observed during xylose fermentation, suggesting that the CO_2_ generated by pyruvate decarboxylase was partially re-assimilated through the synthetic reductive PPP (Xia et al., 2017). Results demonstrated that the net ethanol production of engineered yeast had increased 10%, and the byproducts decreased 11%, demonstrating that the introduction of the PRK-Rubisco route achieved CO_2_ recycling (Xia et al., 2017). An improved dual-module system was constructed in *S. cerevisiae* by employing mutants with alternate cofactor preference. Moreover, PRK and Rubisco from *Ralstonia eutropha* H16 were co-expressed in yeast (Li et al., 2017). *R. eutropha* H16 Rubisco belongs to type I, which is composed of 8 large subunits and 8 small subunits. To assist with proper folding of proteins, the endogenous chaperones of *S. cerevisiae* (Hsp60-HSP10) were co-expressed. Results showed that the productivity of ethanol was 15% higher than in the control strain, and the CO_2_ fixation rate reached 336.6–436.3 mg CO_2_/(L h) with consuming 3.1 g/L total sugar, which was significantly higher than previous natural or engineered microorganisms [5.8–147.0 mg CO_2_/(L h)]. Of note, this experiment proves that type I Rubisco has higher carboxylation activity than type II, likely because the small subunit has the ability to enrich CO_2_, which increases the concentration of CO_2_ at the active site of the enzyme (Li et al., 2017). In the above studies, the introduction of Rubisco and PRK into brewer’s yeast to achieve *in situ* fixation of CO_2_ in the process of bioethanol production and increase the output of the target product bioethanol has laid the foundation for the production of other fuels and chemicals from lignocellulose.

## Introduction of Synthetic Co_2_ Fixation Pathways Into Heterotrophic Microorganism

Despite their naturally existing diversity, the application of CO_2_ fixation pathways for biomanufacturing valuable compounds directly from CO_2_ has been limited so far. These naturally occurring CO_2_ assimilation mechanisms contribute to cell growth rather than to products of interest. Moreover, CO_2_-fixing efficiency of these natural or ALE routes is still unsatisfactory and most of them are inactivated in the presence of oxygen. The emerging field of synthetic biology facilitates to creation of artificial synthetic CO_2_ fixation pathways.

With the goal of enhancing CO_2_ assimilation efficiency, much interest has been devoted to creating stoichiometrically and thermodynamically feasible routes. Starting with CO_2_ capturing enzymes, oxygen-insensitive and kinetically superior carboxylases or reductases have been identified, for instance, PEP carboxylase (EC 4.1.1.31) (Durall et al., 2020), pyruvate decarboxylase (EC 6.4.1.1) (Bar-Even et al., 2010), CoA-dependent carboxylase and metal-dependent FDH (Schwander et al., 2016; Cotton et al., 2018). Next, a series of CO_2_ fixation pathways were predicted *in silico* by evaluating the stoichiometric and thermodynamic feasibility of theoretical pathways, from which to recruit a route characterized by high energy-efficiency. To sustain the proposed draft, potential enzymes were selected from the natural enzyme pool and then engineered to improve their kinetic profiles through directed evolution or rational design. Finally, a cocktail containing all of these enzymes was developed to investigate the feasibility of the proposed pathways *in vitro*, and further, all of these enzymes were introduced into heterotrophic model microorganisms to create artificial autotrophy. ^13^C-labeling method is a powerful tool to pinpoint carbon flow. The ultimate aim is to produce biofuels and value-added compounds directed from CO_2_ through the developed artificial autotrophy.

The first attempt of *de novo* design of a CO_2_ fixation pathway *in vitro* was the crotonyl–coenzyme A (CoA)/ethylmalonyl-CoA/hydroxybutyryl-CoA (CETCH) cycle. Under aerobic condition, one molecule of glyoxylate was generated from two molecules of CO_2_ consuming two molecules of ATP and three molecules of NADPH though this completely artificial carbon fixation pathway with 12 core reactions (Schwander et al., 2016). ECRs with high catalytic efficiency toward CO_2_ were used as the initial enzyme of the entire cycle (Δ_*r*_Gm′ = −26.1 kJ/mol). The methylsuccinyl-CoA dehydrogenase (Mcd) was a rate-limiting enzyme, and a protein engineering approach was used to convert Mcd into a methylsuccinyl-CoA oxidase (Mco). A thermodynamically challenging reaction (Δ_*r*_Gm′ ≥ 10 kJ/mol) is catalyzed by 4-hydroxybutyryl-CoA synthetase in this cycle. Compared to other aerobic naturally occurring CO_2_ fixation flux, the CETCH process consumed the least amount of ATP. The feasibility of the CETCH cycle *in vitro* is a big breakthrough, demonstrating that more efficient CO_2_ assimilation can be realized by rewiring natural elements. However, the final product of this cycle is glyoxylate rather than acetyl-CoA, which hinders the efficient connection between the CETCH cycle and central metabolism. Moreover, it is not economical to employ a complex enzyme assembly to synthesize useful products from CO_2_ on a large scale. The challenges of introducing this artificial synthetic pathway into a heterotrophic host involve the complex interplay among these enzymes used in the CETCH cycle and aboriginal enzymes, as well as this route and endogenous metabolism network. Metabolic regulation also influences expression levels of each enzyme. Last but not least, it is crucial to maintain an efficient supply of reducing power for CO_2_ fixation.

There are several different strategies to activate CO_2_ with specific enzymes in nature. In addition to carboxylation, reduction can also be used to convert CO_2_ to formate by another kind of carbon capturing enzyme, which is FDH. It catalyzes the reversible reaction between CO_2_ and formate, taking part in various metabolic pathways with a variety of redox partners in different subcellular locations (Maia et al., 2017). Roughly, FDH can be divided into two classes, including NAD-independent and NAD-dependent (Jormakka et al., 2003). NAD-independent FDH is characterized by high activity toward CO_2_ and sensitivity to oxygen (Maia et al., 2016; Yu et al., 2017). This class of FDH contains complex redox-active centers harboring different transition metals, such as molybdenum (Mo), tungsten and nonhaemiron, molybdopterin guanine dinucleotide (MGD) and selenocysterine (Niks and Hille, 2019; Lemaire et al., 2020). It has been identified only in prokaryotic organisms. In contrast, NAD-dependent FDH has relatively low activity toward CO_2_ and is insensitive to oxygen (Hartmann and Leimkuhler, 2013; Choe et al., 2014; Altas et al., 2017). This kind of FDH has no metal ions or other redox-active centers and is widely distributed in bacteria, yeasts, fungi, and plants (Maia et al., 2017). However, the conversion of CO_2_ to formate is unfavorable (Δ_*r*_Gm′ = +18.0 kJ/mol) (Liu et al., 2020).

After reducing CO_2_ to formate, the next stage of carbon fixation can be implemented via natural or synthetic formate assimilation pathways (Bar-Even, 2016). Two well-known natural enzymes are capable of activating formate, oxygen-tolerant formate-tetrahydrofolate ligase (FTL) and oxygen-sensitive pyruvate formate-lyase (PFL) (Zelcbuch et al., 2016; Cotton et al., 2018). Starting from these entry-points, two types of formate assimilation pathways proceed, termed the reductive glycine (rGly) pathway and the PFL-PKT cycle (Cotton et al., 2018). Within the aerobic rGly process, formate is attached to THF to form formyl-THF, which can be subsequently reduced to methylene-THF. Glycine is formed by attaching another CO_2_ molecule to methylene-THF via glycine cleavage/synthase system (GCS). Next, serine is generated by adding another methylene-THF molecule. Last, serine is reduced to pyruvate for biomass production. The overall thermodynamics of the pathway starting from formate to serine favor the reductive direction with Δ_*r*_Gm′ = −6 kJ/mol (Yishai et al., 2018). During the anaerobic PFL-PKT cycle, PFL catalyzes the reaction of acetyl-CoA and formate to yield pyruvate. This intermediate is transformed to sugar-phosphates though gluconeogenic and pentose-phosphate pathways. Phosphoketolase (PKT) catalyzes the generation of acetyl-phosphate (AcP) from xylulose 5-phosphate. Finally, acetyl-CoA is regenerated from AcP by the action of phosphate acetyltransferase (PTA, EC 2.3.1.8), and the cycle is closed.

As mentioned above, the rGly pathway is an efficient synthetic route for aerobic assimilation of formate. In *E coli*, foreign enzymes required for the rGly process were expressed, including formate-THF ligase, 5,10-methenyl-THF cyclohydrolase (Fch) and 5,10-methylene-THF dehydrogenase (MtdA) from the *Methylobacterium extorquens* GCS. The resultant strain could biosynthesize cellular glycine and C1 compounds derived from formate and CO_2_ when cells grown under heterotrophic condition (Yishai et al., 2018). Unlike the CBB cycle, the rGly process has few overlaps with cellular central metabolism, reducing the influence of metabolic regulations. Bang and Lee (2018) introduced this pathway in *E. coli* to realize one-carbon assimilation *in vivo* by overexpressing enzymes related to the rGly pathways as well as NAD-dependent FDH for producing reducing power from formate. After glucose depletion, the engineered strain maintained a slight growth using only formate and CO_2_ as substrates, confirming its feasibility for supplying energy and reducing power via NAD-dependent FDH oxidative activity. Based on these pioneer studies, an outstanding work made an attempted to endow *E. coli* grown on formate or methanol and CO_2_ without any other organics via the rGly route. Enzymes in rGly pathways, as well as NAD-dependent FDH, were overexpressed in the strain, in which formate was the only source for cell growth. To improve growth rate, a short-term laboratory evolution was performed. After 40 generations of culture, the doubling time of cells was reduced by 6–8-fold. Genome sequence analysis of the initial and evolved strains revealed two mutations involving energy and reducing power supply (Kim et al., 2020). In another study, cell growth was achieved when integrating the formate assimilation pathway and subsequent ALE without FDH supplying NADH. Genomic sequence analysis showed that mutations covered rGly pathway related genes, biofilm formation genes and formate-utilizing genes (Kim et al., 2019). However, in above studies, the net CO_2_ emission was inevitable since one molecule of CO_2_ was lost during the process of oxidizing formate to supply NADH.

In addition to ethanol, *S. cerevisiae* also has the ability to tolerate high levels of formate in the environment. Moreover, this host harbors necessary genes for the rGly route. Given these unique features, overexpression of rGly route native enzymes in *S. cerevisiae* resulted in net production of glycine using formate as feedstock. However, the addition of sugar instead of formate supported cell growth (de la Cruz et al., 2019). Engineering downstream biomass formation from glycine and subsequent ALE holds promise for use in the development of yeast that are fully autotrophic.

Compared to formate, the more active feature allows formaldehyde easier access to the central metabolism. Therefore, the reduction of formate to formaldehyde coupled with subsequent the natural or synthetic formaldehyde assimilation pathway is an attractive bypass to fix one-carbon compounds. With the goal of shorting the process of one-carbon utilization and accelerating growth starting from formate, an artificial synthetic route was computationally designed, the formolase (FLS) pathway. In this linear route, two natural enzymes with considerable activity toward substrate analogs were identified. Acetyl-CoA synthase (ACS) derived from *E. coli* catalyzes the ATP-dependent conversion of formate into formyl-CoA, and then putative acylating aldehyde dehydrogenase (ACDH) from *Listeria monocytogenes* catalyzes the NADH-dependent reduction of formyl-CoA to formaldehyde. Next, a novel enzyme FLS, which catalyzes the carboligation of three molecules of formaldehyde into one molecule of dihydroxyacetone was created through rational protein design and site-directed mutagenesis based on benzaldehydelyase (BAL) from *Pseudomonas fluorescens biovar I*. Finally, this C3 product can flow into central metabolism. It is speculated by *in silicon* calculations that this completely new synthetic pathway is superior to any natural one-carbon utilization starting from formate pathways due to having the fewest steps and the highest chemical driving force under fully aerobic conditions (the total Gibbs energy change Δ_*r*_Gm′ from formaldehyde to acetyl-CoA is −110.2 kJ/mol). However, the low enzymatic activity of FLS resulted in undetectable cell growth with formate as a substrate (Siegel et al., 2015), suggesting the biotransformation of C1 directly to C3 is difficult to achieve. Given these findings, investigators wondered whether it was possible to produce C2 directly from C1. Recently, a synthetic aerobic acetyl-CoA (SACA) pathway was designed and constructed wherein two molecules of formaldehyde were transferred into one molecule of acetyl-CoA through only three steps (Δ_*r*_Gm′ is −96.7 kJ/mol). First, formaldehyde was condensed into glycolaldehyde (GALD) by glycolaldehyde synthase (GALS). Then, GALD was converted into AcP by acetyl-phosphate synthase (A EC 4.1.2.9). GALS and ACPS were screened and engineered to enhance their catalytic efficiency toward their new substrates, respectively. Finally, the phosphate group of AcP was replaced with CoA catalyzed by PTA, and acetyl-CoA was successfully produced both *in vitro* and *in vivo* (Bar-Even et al., 2010; Li et al., 2019). Although the SACA process is characterized by carbon-conserved and ATP-independent processes, achieving high-efficiency of the pathway has been challenging, likely due to the toxicity of formaldehyde to cells and kinetic bottlenecks of enzymes. To address this issue, renovating the host and implementing further protein engineering may represent promise solutions.

## Reduction of Co_2_ Loss in Microorganisms

In heterotrophic hosts, fixed carbon flows into the central metabolism for synthesis of metabolites for cell growth, during which carbon inevitably suffers great or small loss. For example, the product dihydroxyacetone of the FLS pathway can be converted into acetyl-CoA via the glycolysis pathway with carbon loss and a theoretical carbon yield of 66.7%. To address this issue, significant efforts have been devoted to designing an artificial bypass to reduce the loss of carbon during acetyl-CoA formation. The nonoxidative glycolysis (NOG) pathway achieved biosynthesis of acetyl-CoA from sugar without carbon loss by rewiring the known carbon rearrangement pathway (Bogorad et al., 2013). In this pathway, bifunctional phosphoketolase (Fxpk) from *Bifidobacterium adolescentis* breaks down three molecules of fructose 6-phosphate (F6P) into three molecules of AcP and three molecules of erythrose 4-phosphate (E4P). The three molecules of E4P regenerate two molecules of F6P through carbon rearrangement. The net reaction includes one molecule of F6P that produces three molecules of AcP without carbon loss. By overexpressing Fxpk and removing succinic acid, lactic acid, ethanol, formic acid, and other competitive pathways, the engineered *E. coli* strain produces acetic acid with a yield of 2.2 mol/mol from xylose, close to the theoretical maximum yield (2.5 mol/mol) and exceeding the maximum theoretical value of producing acetic acid from xylose though the EMP pathway (1.67 mol/mol). However, this pathway itself cannot support the growth of cells in a minimal medium that uses sugar as a carbon source, and requires the assistance of reducing equivalents and metabolites in the EMP pathway. To overcome this challenge, researchers further constructed an *E. coli* strain that does not use EMP for glycocatabolism. The engineered strain, which contained 11 overexpressed genes, 10 deleted genes, and more than 50 gene mutations, including three overall regulatory factors, was obtained through directed evolution. This strain can grow in the medium containing glucose, and the carbon conversion rate of anaerobic fermentation of glucose to acetic acid is close to 100% (Lin et al., 2018). By combining the ribulose monophosphate (RuMP) and NOG pathways, acetyl-CoA was produced from methanol through the methanol condensation cycle (MCC) pathway (Bogorad et al., 2014). The first step in MCC is the oxidation of methanol to formaldehyde. This C1 compound and Ru5P were converted to F6P via upstream of the RuMP pathway. Next, acetyl-CoA is produced from F6P by downstream of NOG bypass. Meanwhile, Ru5P was regenerated though the RuMP pathway. Throughout the entire pathway, phosphates were conserved, and C1 compounds were assimilated into acetyl-CoA in an ATP-independent manner. However, construction of MCC *in vivo* has not been implemented, and protein engineering of key enzymes is expected to accelerate carbon flux.

In addition to the EMP pathway, a high level of carbon loss also occurs within TCA cycle. In glyoxysome of plants, the glyoxylate shunt (GS) shares several common intermediates with the TCA cycle. GS involves the conversion of fatty acid to sugar, and the net reaction includes two molecules of acetyl-CoA producesing one molecule of succinic acid without carbon loss. It can be assumed that the reverse version of the glyoxylate shunt (rGS) might be used to generate acetyl-CoA to circumvent loss of carbon. Based on the rGS, a synthetic pathway was designed, in which malate and succinate were converted to oxaloacetate and two molecules of acetyl-CoA. However, the driving force of key steps relies on the hydrolysis of ATP, and the growth rate of the resultant strain was relatively slow (Mainguet et al., 2013). Like the abovementioned C4 metabolites, acetyl-CoA can also be produced from C3 metabolites. A synthetic malonyl-CoA-glycerate (MCG) pathway has been demonstrated to be an efficient way to produce acetyl-CoA (Yu et al., 2018). First, two molecules of PEP were carboxylated into two molecules of oxaloacetate by inputting two molecules of bicarbonate. This reaction was catalyzed by PEP carboxylase, an attractive CO_2_ fixing enzyme regarding its robustness and activity. Oxaloacetate was reduced to malate and then activated to malyl-CoA with ATP consumption. Two molecules of malyl-CoA were split into two acetyl-CoAs and two glyoxylates. The latter products were regenerated to PEP via the glyoxylate assimilation route, during which one CO_2_ was released. Thus, coupled with the CBB cycle, the MCG pathway can fix two molecules of CO_2_ to produce one molecule of acetyl-CoA with the consumption of 5.5 molecules of ATP equivalents and 4 molecules of reducing equivalents. The feasibility of the MCG pathway has been demonstrated both *in vitro* and *in vivo*. Moreover, when coupled with the CBB cycle in cyanobacteria, both acetyl-CoA level and bicarbonate assimilation rate increased one-fold.

**FIGURE 2:**
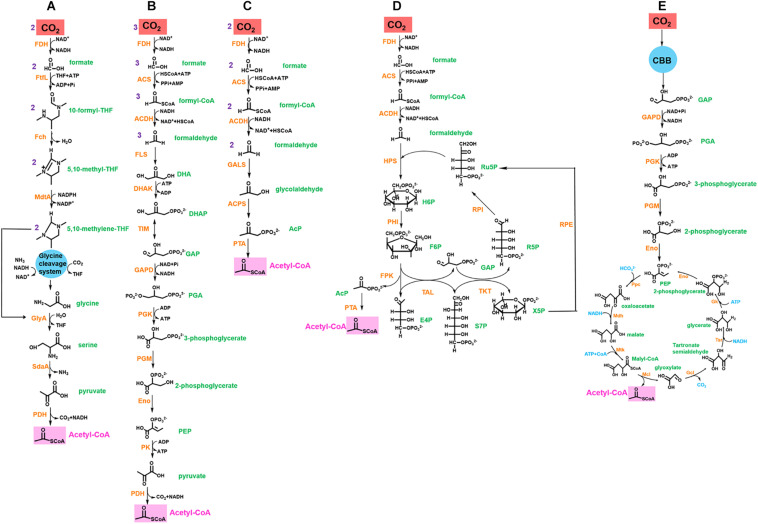
Synthetic CO_2_ fixation pathways for acetyl-CoA synthesis. **(A)** rGly pathway coupled FDH. **(B)** Formolase pathway coupled FDH. **(C)** SACA pathway coupled FDH and partial formolase path. **(D)** MCC pathway coupled FDH and partial formolase path. **(E)** MCG cycle coupled CBB cycle. Abbreviation for enzymes (orange in color): FDH, formate dehydrogenase; FtfL, formate-THF ligase; FchA, 5,10-methenyl-THF cyclohydrolase; MtdA, 5,10-methenyl-THF dehydrogenase; GlyA, serine hydroxymethyl-transferase; SdaA, serine deaminase; PDH, pyruvate dehydrogenase; ACS, acetyl-CoA synthase; ACDH, acetaldehyde dehydrogenase; FLS, formolase; DHAK, dihydroxyacetone kinase; TIM, triose phosphate isomerase; GAPD, glyceraldehyde-3-phosphate dehydrogenase; PGK, 3-phosphoglycerate kinase; PGM, phosphoglycerate mutase; Eno, enolase; PK, pyruvate kinase; GLAS, glycolaldehyde synthase; ACPS, acetyl-phosphate synthase; PTA, phosphate acetyltransferase; HPS, hexulose-6-phosphate synthase; PHI, phosphohexulose isomerase; FPK, fructose-6-phosphate phosphoketolase; TAL, transaldolase; TKT, transketolase; RPE, ribulose 5-phosphate epimerase; RPI, ribose-5 phosphate isomerase; Ppc, phosphoenolpyruvate carboxylase; Mdh, malate dehydrogenase; Mtk, malate thiokinase; Mcl, malyl-CoA lyase; Gcl, glyoxylate carboligase; Tsr, tartronate semialdehyde reductase; Gk, glycerate kinase. Abbreviation for metabolites (green in color): DHA, dihydroxyacetone; DHAP, dihydroxyacetone phosphate; GAP, glyceraldehyde 3-phosphate; PGA, 1,3-biphosphoglycerate; PEP, phosphoenolpyruvate; AcP, acetyl phosphate; H6P, hexulose-6-phosphate; Ru5P, ribulose-5 phosphate; F6P, fructose-6-phosphate; R5P, ribose-5 phosphate; E4P, erythrose-4 phosphate; S7P, sedoheptulose-7 phosphate; X5P, xylose-5 phosphate.

Therefore, the novel synthetic pathway can be designed by coupling CO_2_-fixing reactions with the abovementioned carbon loss-reducing pathways to improve CO_2_ fixation efficiency (Figure 2 and Table 1). Once a source of reducing power is provided, this pathway could theoretically allow growth with CO_2_ as the sole carbon source.

**TABLE 1:** Comparison of synthetic CO_2_ fixation pathways with natural ones.

Pathway	Status	Fixed CO_2_ or HCO${}_{3}^{-}$	Product	ATP consumption	NAD(P)H consumption	CO_2_ capturing enzyme	Specific activity μ mol/min/mg	References
CBB	Natural	3	Glyceraldehyde-3-phosphate	9	6	Ribulose-1,5-bisphosphate, Carboxylase/oxygenase	3.5	Bar-Even et al., 2010
3HP bicycle	Natural	3	Pyruvate	7	5	Acetyl-CoA carboxylase, propionyl-CoA carboxylase	18, 30	Herter et al., 2001; Bar-Even et al., 2010
3HP/4HB cycle	Natural	2	Acetyl-CoA	6	4	Acetyl-CoA carboxylase, propionyl-CoA carboxylase	18, 30	Berg et al., 2007; Bar-Even et al., 2010
rTCA	Natural	2	Pyruvate	2	5	2-oxoglutarate synthase, isocitrate dehydrogenase	–, 53	Kim et al., 1992; Martinez et al., 2007
Wood-Ljungdahl	Natural	2	Acetyl-CoA	1	4	NAD-independent formate dehydrogenase, CO dehydrogenase/acetyl-CoA synthase	2.34, 0.46	Ragsdale, 1997
DC/HB cycle	Natural	2	Acetyl-CoA	5	4	Pyruvate synthase, PEP carboxylase	−, 35	Huber et al., 2008; Garcia-Gonzalez and De Wever, 2017
CETCH	Synthetic	2	Glyoxylate	1	4	CoA-dependent carboxylase		
rGly	Synthetic	3	Pyruvate	2	3	Glycine cleavage system		
Formolase path	Synthetic	3	Dihydroxyacetone phosphate	4	3	NAD-independent formate dehydrogenase		
Partial formolase path + SACA path	Synthetic	2	Acetyl-CoA	2	2	NAD-independent formate dehydrogenase		
partial formolase path + MCC path	Synthetic	1	Acetyl-CoA	1	1	NAD-independent formate dehydrogenase		
CBB + MCG path	Natural + synthetic	2	Acetyl-CoA	5.5	4	PEP carboxylase, ribulose-1,5-bisphosphate, carboxylase/oxygenase		

## The Conversion of Microorganisms From Hemiautotrophy Into Fully Autotrophs

**FIGURE 3:**
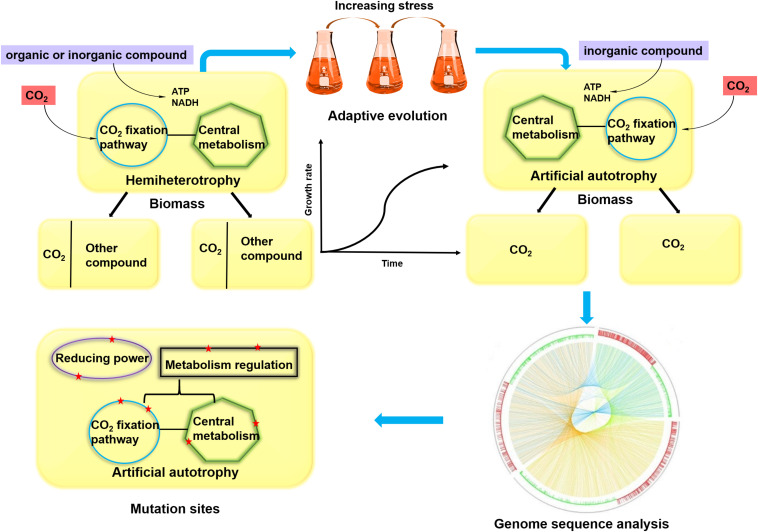
The conversion process of hemiheterotrophy to artificial autotrophy. In hemiheterotrophic microorganism, CO_2_ was fixed into organic carbon via introducing CO_2_ fixation pathway and organic or inorganic compound provided reducing power and energy. Biomass was derived from CO_2_ and another compound. The artificial autotrophic microorganism can be obtained through ALE strategy, in which inorganic compound provided reducing power and energy and biomass was completely derived from CO_2_. To understand the genetic basis for adaptation to autotrophy, genomic analysis can be carried out, and mutation sites were related to CO_2_ fixation pathway, central metabolism, reducing power and metabolism regulation (depicted by red star).

Challenges in rewiring hemiautotrophy to complete autotrophy for steady-state growth on CO_2_ as the sole source of carbon include (1) replacement of the native sugar transport system with a foreign CO_2_ transport system, (2) deletion of part of the sugar metabolism pathway, (3) integration of systems for CO_2_ fixation and central metabolism, (4) supply of reducing power, and (5) adaption of the strain to grow under this rewired metabolism. Fully considering these criteria, completely autotrophic microorganisms have been developed (Gleizer et al., 2019; Gassler et al., 2020) (Figure 3). For *E. coli*, carbonic anhydrase (CA, which catalyzes the reversible reaction between CO_2_) was used to concentrate and transport CO_2_ into cells. Key enzymes in the CBB cycle were heterologously expressed (Rubisco and PRK), whereas genes related to glycolysis and the oxidative pentose-phosphate pathway were knocked out. Importantly, NAD-dependent FDH was introduced into cell to generate NADH from formate. NADH provides the reducing power to drive carbon fixation and serves as the substrate for ATP generation via oxidative phosphorylation. Although three necessary enzymes were introduced into *E. coli*, these efforts failed to generate a complete autotrophy due to the complexity of native metabolism and regulation networks in cells. Thus, long-time ALE was performed to redistribute the central metabolic flux. Finally, a complete autotrophic strain was obtained after 350 days of evolving. Genomic sequence analysis showed that CBB cycle-related genes, genetic selective pressure-related genes and nonfunctional genes were mutated (Erb et al., 2019; Gleizer et al., 2019). However, one molecule of CO_2_ was inevitably lost during the process of oxidizing formate to supply NADH, resulting in a net CO_2_ emission under autotrophic conditions.

As a model eukaryotic microbe, *Pichia pastoris* is also regarded as a proper chassis to construct autotrophs. The CBB cycle was introduced into cells though rewriting the xylulose monophosphate cycle and deleting methanol assimilation genes in peroxisomes. Energy and reducing power were provided by methanol oxidation. The maximum specific growth rate on CO_2_ increased more than two-fold via adaptive evolution. Genomic sequence analysis showed that mutations were involved in CBB cycle related genes, NADH and ATP synthesis genes. These results indicated the importance of carbon fixation pathways, energy and reducing power supply on cell growth using CO_2_ as a sole carbon source (Gassler et al., 2020). However, similar with employing formate as energy and reducing power source, the oxidation of methanol also resulted in the net emission of CO_2_.

## Conclusion and Perspectives

Efforts to implement CO_2_ fixation metabolism into heterotrophic microorganisms have not only focused on the well-known CBB cycle. Other naturally occurring pathways, as well as synthetic routes, have also been successfully introduced into heterotrophic hosts. Recent advances in the conversion of heterotrophic metabolism to fully autotrophic growth marked a new period of “artificial autotrophy.” However, net CO_2_ assimilation has not yet been demonstrated since fixed CO_2_ is lost again during reducing power and energy generation. Sustainable production of biofuels and value-added compounds will become possible only if artificial autotrophy can efficiently fix CO_2_ without carbon loss. Future efforts to develop artificial autotrophy are discussed in this section.

First of all, to achieve autotrophic growth, sustainable sources of energy and reducing equivalents are vital. Introducing light-cycle reactions into heterotrophic hosts remains challenging due to their extremely complex nature. Artificial photoautotrophy might be realized by introducing a fully functional proteorhodopsin photosystem into model hosts or hybridizing light-capturing nanoparticles with cells (Martinez et al., 2007; Guo et al., 2018). However, these photosynthetic biological hybridization systems are still in the early stages of development. Other challenges also require continuous research and exploration, such as biocompatibility of materials, selection of light collection devices, and seamless coupling of biological and nonbiological components (Cestellos-Blanco et al., 2020). In addition to light energy, electrical energy can also be used to produce energy carriers, such as formate, hydrogen, carbon monoxide, methanol, methane, and so on (Chen H. et al., 2020). In a pioneer study, a genetically engineered autotrophic microorganism, *Ralstonia eutropha* H16, produced higher alcohol levels in an electric bioreactor that used CO_2_ as the sole carbon source and electricity as the sole energy input. The CO_2_ on the cathode driven by electricity was reduced to formic acid, which was then converted into isobutanol and 3-methyl-1-butanol. This process integrated CO_2_ fixation, electrochemical formic acid formation, and higher alcohol synthesis, opening up possibilities for electrically driving CO_2_ to commercial chemicals (Li et al., 2012). Although this work proves the feasibility of electrochemical fixation of CO_2_, the lack of a full understanding of the host and the production of hydrogen peroxide during the production process affected cell growth, limiting continuous improvement of the system. Enzymatic catalysis is a powerful platform to drive CO_2_ fixation, such as NAD-dependent FDH and hydrogenase (Antonovsky et al., 2017). The former has been successfully applied in construction of artificial autotrophy via the CBB cycle as described above. However, carbon loss is inevitable during the generation of NADH. Therefore, hydrogenase is a promising option for providing energy and reducing equivalents without carbon loss (Yu, 2018). In this context, the (an)aerobic fermentation of synthesis gas (syngas) (H_2_/CO/CO_2_) and industrial off-gases has been proven to be an attractive platform for fixing CO_2_ to produce a variety of chemicals in some natural autotrophic microorganisms, including acetogenic bacteria (*Clostridium autoethanogenum* and *Clostridium ljungdahlii*) (Mock et al., 2015; Emerson et al., 2019), *Cupriavidus necator* (Garcia-Gonzalez and De Wever, 2017) and *Oligotropha carboxidovorans* (Siebert et al., 2020), in which carbon can be fixed via CBB cycle or the Wood-Ljungdahl pathway with hydrogen as energy source. Similarly, Keller et al. (2013) expressed five genes of subpathway 1 in 3-HP/4-HB cycle from the thermophilic bacteria *Metallosphaera sedula* in *Pyrococcus furiosus*. The engineered strain used hydrogen as the electron donor to convert CO_2_ and acetyl-CoA into the valuable chemical 3-hydroxypropionic acid (Keller et al., 2013).

Second, CO_2_ fixation pathways are the key component of artificial autotrophy. As described above, although the CBB cycle is ubiquitous in the biosphere, carbon yield is lower and energy consumption is higher. Moreover, other natural aerobic CO_2_ assimilation routes involve too many enzymes, for instance 3-HP bicycle. Hence, a synthetic pathway with higher carbon yield, reduced energy cost and relatively fewer enzymes is a more attractive candidate for reconstruction of artificial autotrophy. In this respect, strong advances in synthetic biology have enabled the rational design of a novel CO_2_ fixation pathway based on a variety of established routes reported over the last decade. For example, the formate assimilation pathway has been engineered in model microorganisms, and an entire metabolic pathway can be developed by coupling upstream CO_2_ capturing and downstream production of acetyl-CoA and other metabolites for cell growth and product biosynthesis. CO_2_ can be reduced to formate by the action of NAD-independent FDH or electrodes, and the recruitment of SACA bypasses downstream pathways and can efficiently produce acetyl-CoA without any carbon loss. Moreover, the influence of carbon flux regulation on CO_2_ assimilation metabolism will be dramatically minimized due to few overlaps between exogenous metabolism and the endogenous cellular metabolic network. In addition to well-studied CO_2_ capturing enzymes, such as Rubisco or NAD-independent FDH, more novel and efficient enzyme candidates in the CO_2_ fixation pathway might be identified using advanced technologies for DNA sequencing, bioinformatics and structure-function predictions. Recently, a CO_2_-reducing formate dehydrogenase complex (FdhAB) was identified from environmental samples by genome-resolved metagenomics (Figueroa et al., 2018). Moreover, the rate-limiting enzyme in CO_2_ fixation pathways may have to be evolved or engineered with new features or improved kinetic properties.

Third, in most reactions, increased substrate concentrations can improve thermodynamics and enzyme conversion efficiency, as well as reducing enzyme side reaction activity. CO_2_ capture mechanisms can be used to boost CO_2_ concentration, including transmembrane bicarbonate pumps, transport proteins, carbonic anhydrase and microcompartments (Kerfeld and Erbilgin, 2015). Functional expression of foreign carboxysome in *E. coli* laid the groundwork for CO_2_ condensation and fixation (Bonacci et al., 2012; Gong et al., 2015). Moreover, this spatial organization provides more stable enzyme structures, facilitates substrate channeling between active sites, and promotes carbon flux in a desirable direction (Siu et al., 2015).

Last but not least, the selection of a proper host plays a crucial role in the development of artificial autotrophy. A variety of criteria should be taken into account, including tolerance of feedstock, culture conditions, products of interest, cell growth rate, robustness, feasibility and stability of genetic manipulation. Compared to *E. coli*, *S. cerevisiae* has a higher tolerance for formate or other toxic substrates, as well as more endogenous carbon anabolic enzymes. From a biotechnology perspective, these features make *S. cerevisiae* a very promising chassis for the CO_2_-fixing bio-industry.

Although all of abovementioned essential components for autotrophy are introduced into a suitable host, the constructed artificial autotrophic microorganism cannot grow solely on CO_2_ since it is “accustomed” to heterotrophy. Therefore, this engineered strain should be adapted to be “fed” with CO_2_. In recent studies, autotrophic growth was achieved by use of an ALE approach, and many essential mutation sites were identified, involving the CO_2_ fixation pathway, central metabolism, reducing power and metabolic regulation (Figure 3 and Table 2). These sites can be used for modified targets in future work.

**TABLE 2:** Essential mutations for adaptation to autotrophy.

Overexpression enzymes	Energy source and reducing power	Mutated genes	Functions	Host	References
Ribulose-1,5-bisphosphate, carboxylase/oxygenase, phosphoribosyl kinase, formate dehydrogenase	Pyruvate	*prs* (ribose-phosphate diphoskinase), *pgi* (glucosephosphate isomerase), *serA* (3-phosphoglycerate dehydrogenase)	CBB cycle	*E. coli*	Herz et al., 2017
		*crp* (cAMP receptor protein), *ppsR* (PEP synthetase regulatory protein)	Metabolism regulation		
Formate-THF ligase, methenyl-THF cyclohydrolase, methylene-THF dehydrogenase, serine glyoxylate transaminase, serine hydroxymethyltransferase hydroxypyruvate reductase, glycerate kinase	Formate	*metF* (methylenetetrahydrofolate reductase) *purU* (formyltetrahydrofolate deformylase)	Folate metabolism	*E. coli*	Kim et al., 2019
		*purT/purN* (phosphoribosylglycinamide formyltransferase) *hycA/fnr* (formate hydrogenlyase)	Formate hydrogen lyase regulation		
Ribulose-1,5-bisphosphate, carboxylase/oxygenase, phosphoribosyl kinase, formate dehydrogenase, carbonic anhydrase	Formate	*prs* (ribose-phosphate diphoskinase), *pgi* (glucosephosphate isomerase), *aroH* (2-dehydro-3-deoxyphosphoheptonate aldolase)	CBB cycle	*E. coli*	Gleizer et al., 2019
		*fdh* (formate dehydrogenase)	Reducing power		
Phosphoribosyl kinase phosphoglycerate kinase glyceraldehyde-3-phosphate dehydrogenase triosephosphate isomerase transketolase	Methanol	*Prk* (phosphoribosyl kinase)	CBB cycle	*P. pastoris*	Gassler et al., 2020
		*NMA1* (nicotinic acid mononucleotide adenylyltransferase)	Reducing power		
Formate-THF ligase, methenyl-THF cyclohydrolase, methylene-THF dehydrogenase, glycine cleavage/synthase system, serine hydroxymethyltransferase, serine deaminase, formate dehydrogenase	Formate	*fdh* (formate dehydrogenase), *pntAB* (membrane-bound transhydrogenase)	Reducing power	*E. coli*	Kim et al., 2020

In conclusion, the development of synthetic biology has provided the possibility of designing efficient biological carbon-fixing processes through understanding the diversity of carbon-fixing organisms and their metabolic pathways in nature, as well as the exploration of efficient carbon-fixing elements. We believe that CO_2_ fixation by microbial organisms may make a significant contribution to building a sustainable society.

## Author Contributions

BL and JY designed the manuscript. BL and YZ prepared the manuscript. JY polished the manuscript. All authors contributed to the article and approved the submitted version.

## Conflict of Interest

The authors declare that the research was conducted in the absence of any commercial or financial relationships that could be construed as a potential conflict of interest.
