# Corrigendum: Recent Advances in Developing Artificial Autotrophic Microorganism for Reinforcing CO_2_ Fixation

**DOI:** 10.3389/fmicb.2020.632455

**Published:** 2020-12-14

**Authors:** Bo Liang, Yukun Zhao, Jianming Yang

**Affiliations:** ^1^Energy-rich Compounds Production by Photosynthetic Carbon Fixation Research Center, Qingdao Agricultural University, Qingdao, China; ^2^Shandong Key Lab of Applied Mycology, College of Life Sciences, Qingdao Agricultural University, Qingdao, China; ^3^Pony Testing International Group, Qingdao, China

**Keywords:** CO_2_ fixation, autotrophy, heterotrophy, synthetic biology, reducing power, cell factory

An author name was incorrectly spelled as Yunkun Zhao. The correct spelling is Yukun Zhao

The authors apologize for this error and state that this does not change the scientific conclusions of the article in any way. The original article has been updated.

